# Left Ventricular Dilation and Pulmonary Vasodilatation after Surgical Shunt for Treatment of Pre-Sinusoidal Portal Hypertension

**DOI:** 10.1371/journal.pone.0154011

**Published:** 2016-04-27

**Authors:** Orlando Luis de Andrade Santarém, Roberto de Cleva, Flávia Megumi Sasaya, Marianna Siqueira de Assumpção, Meive Santos Furtado, Alfonso Julio Guedes Barbato, Paulo Herman

**Affiliations:** 1 Department of Gastroenterology, University of São Paulo Medical School, São Paulo, São Paulo, Brazil; 2 Department of Radiology, University of São Paulo Medical School, São Paulo, São Paulo, Brazil; Scuola Superiore Sant'Anna, ITALY

## Abstract

**Objective:**

The aim of this study was to prospectively investigate the long-term cardiovascular and pulmonary hemodynamic effects of surgical shunt for treatment of portal hypertension (PH) due to *Schistosomiasis mansoni*.

**Location:**

The University of São Paulo Medical School, Brazil; Public Practice.

**Methods:**

Hemodynamic evaluation was performed with transesophageal Doppler and contrast-enhanced echocardiography (ECHO) on twenty-eight participants with schistosomal portal hypertension. Participants were divided into two groups according to the surgical procedure used to treat their schistosomal portal hypertension within the last two years: group 1—distal splenorenal shunt (DSRS, n = 13) and group 2—esophagogastric devascularization and splenectomy (EGDS, n = 15).

**Results:**

The cardiac output (5.08 ± 0.91 L/min) and systolic volume (60.1 ± 5.6 ml) were increased (p = 0.001) in the DSRS group. DSRS participants had a significant increase (p < 0.0001) in their left ventricular end-systolic and end-diastolic diameters as well as in their left ventricular end-diastolic and end-systolic volumes (p < 0.001) compared with the preoperative period. No statistically significant difference was found in the patients who underwent EGDS. ECHO revealed intrapulmonary vasodilatation (IPV) in 18 participants (64%), 9 DSRS and 9 EGDS (p > 0.05).

**Conclusions:**

The late increase in the cardiac output, stroke volume and left ventricular diameters demonstrated left ventricular dilatation after a distal splenorenal shunt. ECHO revealed a greater prevalence for IPV in patients with schistosomiasis than has previously been described in patients with PH from liver cirrhosis.

## Introduction

*Schistosoma mansoni* is the major infectious agent of schistosomiasis, an endemic disease primarily found in tropical areas and Brazil. According to the World Health Organization, more than 600 million people live in risk areas across 75 countries and approximately 200 million people are infected [1]. Schistosomiasis, in its most severe form, can lead to a presinusoidal portal hypertension with minimal hepatic dysfunction and marked splenomegaly [2,3]. The leading cause of morbidity and mortality in mansonic schistosomiasis is esophageal bleeding from varices rupture [4,5,6], and surgical treatment of portal hypertension is the best therapeutic option [7,8]. Two surgical procedures have been largely employed, distal splenorenal shunt (DSRS) and esophagogastric disconnection with splenectomy (EGDS), inspiring a debate about which technique is better. DSRS is an effective treatment of portal hypertension (with low rebleeding rates), but it has high rates of postoperative portosystemic encephalopathy [9]. EGDS is a relatively simple technique with good results and the absence of postoperative encephalopathy, and it is the treatment of choice for the majority of groups undergoing treatment of schistosomiasis presinusoidal portal hypertension [5,9,10].

Hemodynamic studies of schistosomiasis have shown the important role of splenomegaly. When systemic and hepatic hemodynamics were evaluated [11,12], high cardiac outflow and low peripheral resistance were observed in almost all patients, confirming the existence of hyperkinetic circulation, although the cardiac rate and mean arterial pressure remained normal. Our group has previously shown that this hyperdynamic circulation is corrected by esophagogastric devascularization and splenectomy, and it is maintained after a distal splenorenal shunt [12]. To the best of our knowledge, there are no data on the late postoperative hemodynamic effects of these surgical techniques (shunt x devascularisation procedures).

Moreover, portal hypertension affects other organs and systems beyond the liver, such as the lungs, resulting in pulmonary hypertension or hepatopulmonary syndrome (HPS) [11,12,13,14]. Recently, HPS has been recognized in patients with portal hypertension in the absence of cirrhosis [14,15], portal vein thrombosis [16], Budd-Chiari syndrome [17], and hypoxic hepatitis [18]), as well as in patients with acute and chronic hepatitis in the absence of portal hypertension [19]. Additionally, there are no data on the presence or effects of surgical procedures on intrapulmonary vasodilation (IPV) in schistosomiasis presinusoidal portal hypertension.

The aim of our study was to investigate late postoperative systemic hemodynamic and pulmonary effects (intrapulmonary vasodilatation) after two different surgical procedures for treatment of schistosomiasis presinusoidal portal hypertension.

## Materials and Methods

### Ethical considerations

The study protocol was performed according to the ethical recommendations of the Declaration of Helsinki and was approved by the Ethical Committee of the Hospital das Clínicas, at the University of São Paulo Medical School. The study was conducted with the participants signed consent.

### Participant recruitment

Twenty-eight participants with schistosomal portal hypertension and previous episodes of digestive bleeding due to esophageal variceal rupture who underwent elective surgical treatment within the last two years were prospectively studied. All participants underwent liver biopsy to confirm the diagnosis. Sixteen participants were males and twelve were females; the mean age was 46 years (range 30 to 70 years). Physical examinations were performed to detect orthodoxy, clubbing in the fingers and toes and peripheral cyanosis. The complete blood count, aminotransferases, blood urea nitrogen, creatinine, prothrombin time, partial thromboplastin time, albumin and other routine tests were measured in all participants to exclude concomitant cirrhosis. For hemodynamic studies, participants were divided according to the surgical procedure used to treat their schistosomal portal hypertension between June 1998 and March 2005: group 1 (distal splenorenal shunt—DSRS) and group 2 (esophagogastric devascularization and splenectomy—EGDS). Two radiologists, who were blinded to the type of surgical procedure, performed standard echocardiograms (ECHO), consisting of two-dimensional, M-Mode and Doppler blood flow measurements, using a GE Vivid 7 ultrasound system with a 2.5 MHz transducer for all participants. The results were compared with a previous routine ECHO examination that was performed before surgical treatment. All participants underwent contrast-enhanced echocardiography by infusion of agitated saline solution. In each participant, an intravenous line was placed in the forearm. A forceful hand injection of 10 ml of agitated saline solution was performed while images were simultaneously obtained in the parasternal four-chamber view using a 2.5 or 3.5 MHz phased transducer in a GE Vivid 7 ultrasound system (General Electric, Milwaukee, WI, USA). A positive exam was defined as the appearance of bubbles in the left atrium after 3 cardiac cycles. The appearance of bubbles in the left atrium after 2 cardiac cycles was considered intracardiac shunting [20], and participants with this shunting were excluded from the study. A negative contrast echocardiogram was defined as the absence of bubbles in the left heart chambers. Two cardiologists, who were blinded to the clinical history and arterial blood gas results, independently reviewed the films for each participant. A room air arterial blood gas (ABG) was obtained from the radial artery in participants at rest in the sitting position, and the oxygenation saturation, arterial blood oxygen, and alveolar-arterial O_2_ gradient [D (A-a) O_2_] were evaluated. Determination of the D (A-a) O_2_ was calculated as the difference between the alveolar oxygen pressure (PAO_2_) and the arterial oxygen pressure (PaO_2_), where PAO_2_ = [0.21 x (barometric pressure—47)] − (1.25 × PaCO_2_) [21]. HPS was diagnosed as D (A-a) O_2_ > 15 mmHg [22,23].

A noninvasive evaluation of the systemic hemodynamics was performed in all participants using an esophageal Doppler device (Cardio Q). The results were compared with a control group consisting of ten participants without portal hypertension who were monitored during endoscopic evaluation for dyspepsia. The Cardio Q (Deltex Medical, Irving, TX, USA) esophageal Doppler monitor consists of a continuous-wave Doppler transducer (4 MHz) at the tip of a transesophageal probe connected to a monitor displaying the blood flow velocity profile. Briefly, the probe was gently advanced after oral introduction to the mid-esophagus (approximately 35 cm from the upper dental arcade) and rotated posteriorly to obtain a characteristic aortic blood flow signal [24]. The probe position was optimized to record the peak velocity by slow rotation in the long axis and the depth of insertion to generate a clear signal. The gain setting was adjusted to obtain the best outline of the aortic velocity waveform, and a filter eliminated the noise related to low-frequency vessel wall motion [24]. The stroke volume (SV) was calculated as follows [25]: SV = CSA_Ao_ x K x V_Ao_ (t) dt, where V_Ao_ (t) represents the instantaneous maximum aortic velocity, t represents the cardiac ejection time (the integral of instantaneous maximum velocity during cardiac ejection, representing the stroke distance), CSA_Ao_ represents the cross-sectional area of the descending thoracic aorta (cm^2^), and K represents a correcting factor (= 1.43) whose purpose is to transform the blood flow measured in the descending thoracic aorta into global cardiac output with the assumption that a constant fraction (70%) of the total blood flow passes through the descending aorta [25]. CSA_Ao_ is estimated from a normogram based on the participant’s age, weight and height [26]. The monitor was preset to calculate the cardiac output (CO: L/min) by averaging the stroke volume over 10 beats and multiplying the value obtained by the heart rate.

### Statistical analysis

Statistical analysis was performed using PASW-18 IBM software program (SPSS, Westlands Road, Quarry Bay, Hong Kong). All data were presented as the absolute (n) and relative (%) frequency or means and standard deviations, as appropriate. Statistical analysis was performed using the two-tailed paired t-test for quantitative variables, and the Fisher’s exact test for qualitative variables was used to determine the intergroup differences in numerical data. A p < 0.05 value was considered significant.

## Results

Thirty-two participants were initially enrolled in the study but four were excluded due to the presence of concomitant cirrhosis. There was no significant difference in the age (49.7 ± 14 versus 47.4 ± 11 years; p = 0.64), body surface area (1.7 ± 0.2 versus 1.6 ± 0.1 m^2^; p = 0.10), follow-up time (9.9 ± 4.6 years versus 8.4 ± 2.3 years; p = 0.32) and liver function between the DSRS and EGDS participants. Laboratory data are shown in Table 1.

**Table 1:** Laboratory data of the participants with hepatosplenic schistosomiasis mansoni after surgical treatment for portal hypertension by distal splenorenal shunt (DSRS) and esophagogastric devascularization with splenectomy (EGDS).

Variables	DSRS (n = 13)	EGDS (n = 15)	Normal Values
ALT (IU/L)	31.00 ± 14.10	32,60 ± 13,40	7.00–45.00
AST (IU/L)	40.20 ± 14.90	38.50 ± 14.20	7.00–45.00
GGT (IU/L)	31.07 ± 10.80	37,80 ± 10.30	7.00–50.00
ALP (IU/L)	87.70 ± 22.90	90.40 ± 16.00	60.00–122.00
BUN (mg/dL)	27.30 ± 14.40	25.00 ± 7.00	10.00–50.00
Cr (mg/dL)	0.78 ± 0.18	0.75 ± 0.15	0.60–1.40
TP (g/dL)	7.40 ± 1.10	7.80 ± 0.40	6.00–8.00
ALB (g/dL)	3.91 ± 0.60	4.20 ± 0.40	3.50–5.00
PT (s)	12.90 ± 1.14	12.70 ± 0.70	14.00 ± 2.00
PTT (s)	28.00 ± 4.40	25.00 ± 3.30	30.00 ± 2.00
TB (mg %)	1.61 ± 0.83*	0.73 ± 0.20	1.40
IB (mg %)	1.06 ± 0.69*	0.30 ± 0.10	0.80
Hb (g/dL)	13.30 ± 1.15	13.90 ± 1.70	12.00–18.00
Ht (%)	37.00 ± 5.80	41.40 ± 4.53	36.00–54.00
WBC (10^3^/mm^3^)	4.38 ± 12.40	6.47 ± 1.70	4.00–10.00
PLT (10^3^/mm^3^)	194.00 ± 55.60	258.60 ± 48.40	150.00–400.00

The results are expressed as the mean ± SD.

ALT, alanine aminotransferase; AST, aspartate aminotransferase; GGT, gamma glutamyl transpeptidase; ALP, alkaline phosphatase; BUN, blood urea nitrogen; Cr, creatinine; TP, total serum protein; ALB, albumin; PT, Prothrombin time; PTT, Partial thromboplastin time; TB, Total bilirubin; IB, indirect bilirubin; Hb, Hemoglobin; Ht, hematocrit; WBC, white blood cells; PLT, platelets

*p < 0.01.

The results of transthoracic conventional echocardiogram are summarized in Table 2. No ventricular hypertrophy or segmental contraction abnormality was observed, and none of the participants presented valve lesions or pericardial effusions. The DSRS group presented a significant increase (p < 0.0001) in the left ventricular end-systolic (LVESD) and end-diastolic (LVEDD) diameters compared with the preoperative period, while there were no differences in the EGDS group. The DSRS group also had a significant increase (p < 0.001) in left ventricular end-diastolic (LVEDV) and end-systolic (LVESV) volumes compared with the preoperative period. No significant differences were observed in the EGDS patients. A statistically significant decrease was observed in the ejection fraction (EF; p = 0.006) and shortening fraction (SF; p = 0.03) for participants who underwent DSRS compared with the preoperative period, while EGDS participants did not have any significant differences. A significant increase (p = 0.002) in the left atrium (LA) diameter was also observed in DSRS participants compared with the preoperative period. No differences were observed in the EGDS participants. There was no significant difference in the intraventricular septum (Se) and posterior wall (PW) thickness in the DSRS and EGDS groups compared with the preoperative period.

**Table 2:** Transthoracic echocardiography results in participants with mansonic schistosomiasis before (Preop) and after surgical treatment for portal hypertension by distal splenorenal shunt (DSRS) and esophagogastric devascularization with splenectomy (EGDS).

Variables	Preop (n = 28)	DSRS (n = 13)	EGDS (n = 15)	Normal range
LA (mm)	35.8 ± 6.0	40.0 ± 5.0***	37.0 ± 4.0	20–40
LVEDD (mm)	49.5 ± 5.0	55.5 ± 4.7*	50.0 ± 3.2	35–55
LVEDV (mL)	123.2 ± 35.0	168.5 ± 44.0**	126.9 ± 25.0	50–150
LVESD (mm)	29.5 ± 2.8	36.4 ± 4.0*	32.5 ± 3.0	20–35
LVESV (mL)	26.5 ± 6.9	48.0 ± 16.0**	35.0 ± 8.0	30–50
SF (%)	39.6 ± 1.8	34.6 ± 2.3***	37.9 ± 2.8	30–40
EF (%)	78.3 ± 2.3	70.8 ± 2.7**	72 ± 3.5	65–80
Se (mm)	8.5 ± 0.5	9.1 ± 1.1	8.8 ± 1.8	7–11
PW (mm)	8.8 ± 0.4	8.5 ± 1	8.6 ± 1.7	7–11

The results are express as the mean ± SD.

Preop, patients with portal hypertension due to hepatosplenic mansonic schistosomiasis before surgical treatment of portal hypertension; DSRS, Distal Splenorenal Shunt; EGDS, Esophagogastric Devascularization with Splenectomy; LA, left atrium; LVEDD, left ventricular end-diastolic diameter; LVEDV, left ventricular end-diastolic volume; LVESD, left ventricular end-systolic diameter; LVESV, left ventricular end-systolic volume; SF, shortening fraction; EF, ejection fraction; Se, septum wall thickness; PW, posterior wall thickness

*p < 0.0001

**p < 0.001

*** p < 0.05 between the DSRS and Control groups.

Contrast-enhanced echocardiography revealed intravascular pulmonary vasodilatation (IPV) in 9 DSRS (69%) and 9 EGDS (60%) participants. In spite of the prevalence of IPV, a widening of the arterial oxygen gradient > 15 mmHg (hepatopulmonary syndrome) was observed in only one participant (7.6%). None of the participants presented with orthodoxy, clubbing in the fingers and toes or central or peripheral cyanosis.

Results of the systemic hemodynamic evaluation by esophageal Doppler are presented in Table 3. The cardiac output (5.08 ± 0.91 L/min) was increased (p = 0.001) in the DSRS group, while the EGDS participants presented no difference (p = 0.47) in the cardiac output (4.36 ± 0.59 L/min) compared with the control group (4.17 ± 0.52 L/min). The DSRS participants also presented a significant increase (p = 0.001) in the systolic volume (60.1 ± 5.6 ml) compared with the control group (53.2 ± 5.6 ml), while no significant difference (p = 0.41) was observed in the EGDS group (56.0 ± 9.4 ml). There was no statistically significant difference between the heart rate (EGDS, p = 0.22; DSRS, p = 0.91) and mean arterial pressure (EGDS, p = 0.40; DSRS, p = 0.06) between the EGDS and DSRS groups.

**Table 3:** Late postoperative hemodynamic parameters in participants with portal hypertension due to hepatosplenic mansonic schistosomiasis.

Variables	DSRS(n = 13)	EGDS(n = 15)	Control(n = 10)	Normal Values
HR (beats/min)	83.40 ± 10.40	77.00 ± 10.00	78.00 ± 12.00	80–100
MABP (mmHg)	93.6 ± 12.40	92.40 ± 12.75	91.20 ± 10.35	80–100
CO (L/min)	5.30 ± 0.94*	4.30 ± 0.58	3.95 ± 0.34	5–8
SV (mL/beat)	60.20 ± 5.80*	56.60 ± 9.60	45.00 ± 18.00	40–60

The results are express as the mean ± SD.

DSRS, distal splenorenal shunt; EGDS, esophagogastric devascularization and splenectomy; HR, Heart rate; MABP, mean arterial blood pressure; CO, cardiac output; SV, systolic volume

*p < 0.01 between DSRS and Control.

## Discussion

Hemodynamic changes after surgical treatment for schistosomiasis portal hypertension may improve our understanding of the hyperdynamic circulation observed in patients with presinusoidal portal hypertension and preserved liver function [9,11,12].

The aim of this study was to evaluate the hemodynamic effects on pulmonary circulation in the late postoperative period in patients undergoing surgical repair of portal hypertension by schistosomiasis. there was no randomization techniques and no assessment of mortality from variceal bleeding.

The late hemodynamic effects of different surgical techniques for treating schistosomiasis, such as induced portal hypertension, derivation (DSRS) and disconnection procedures (EGDS), were prospectively evaluated in our study. The groups were similar with respect to age, body surface area and time of follow-up after surgery.

Although a pulmonary artery catheter is considered the standard of care and is the most widely used method for systemic hemodynamic evaluation in intensive care units [27], it is an invasive procedure and presents with significant complications that limit application outside the hospital. Therefore, a minimally invasive technique, such as transesophageal Doppler, which provides lower rates of complications [28,29] and easily obtainable hemodynamic variables [29,30], is desired. Several studies have demonstrated the effectiveness of this technique compared to a pulmonary artery catheter, reporting a strong positive correlation between measures of the cardiac output by both methods in patients undergoing abdominal or cardiac surgery as well as in patients with sepsis under mechanical ventilation [28,29,30,31,32,33]. Nevertheless, no studies have evaluated the use of esophageal Doppler to measure the cardiac output on an ambulatory basis for patients with portal hypertension. Moreover, we compared the results obtained in the portal hypertensive patients with a control group of patients without portal hypertension using the same technique, including sedation.

Portal hypertension determines a hyperdynamic circulation that is characterized by increased cardiac index and decreased systemic vascular resistance. We have previously observed normalization of the cardiac output and systemic vascular resistance in EGDS participants in the immediate postoperative period, while participants undergoing DSRS maintained a hyperdynamic pattern, suggesting a significant contribution of splenic overflow [11]. However, there are no reported data on whether the hemodynamic pattern and its effects on the cardiovascular system remain during long-term follow-up.

In this study, we observed, after nine years of follow-up, a significant increase in the cardiac output and stroke volume in participants who underwent DSRS, while the values for EGDS participants were similar to the control group (p > 0.05). Therefore, after long-term follow-up, participants who underwent DSRS maintained hyperdynamic circulation compared with participants in the EGDS group.

We also observed a significant increase in both the systolic and diastolic diameters of the left ventricle in the DSRS group compared with the immediate preoperative period. The same significant increase was observed for the end-systolic and end-diastolic left ventricular volumes. EGDS participants did not show any change in their systolic or diastolic diameters or left ventricle volumes compared with the preoperative period. These findings demonstrate the volume overload state secondary to the blood flow through the splenorenal anastomosis. In DSRS participants, the splenorenal shunt behaves as a circuit of low pressure and resistance compared with the normal vascular tone, which increases the venous return, volume overload and diastolic dilation of the left ventricle and may progress to congestive heart failure [34]. On the other hand, participants who undergo EGDS maintain normal hemodynamic parameters, which is similar to patients with arterial-venous fistula occlusion [34,35,36]. Myocardial adaptive change depends on the functional reserve of the myocardium and the intensity of the fistula blood flow [34,37]. Therefore, if the increase in the venous return is gradual, as in patients with chronic renal failure who have arteriovenous fistulas, cardiovascular compensatory mechanisms could provide adequate conditions to accommodate the additional volume, maintaining adequate systolic function with left ventricular dilation [23]. In contrast, patients with pressure overload, such as aortic stenosis or arterial blood hypertension, develop left ventricular hypertrophy that is characterized by symmetric hypertrophy of the septum and posterior wall of the left ventricle [34]. The absence of left ventricular hypertrophy observed in our study favors the hypothesis that the volume overload determines left ventricular dilation in DSRS participants.

Volume overload can also increase the stretch of cardiac muscle fibers and, consequently, increase the shortening fraction (SF) [38]. Nevertheless, a significant decrease in SF was observed in the DSRS group compared to the preoperative period. This observation suggests that compensatory mechanisms may be inefficient to maintain adequate systolic function [39], as observed in patients with severe aortic insufficiency, which presents as systolic dysfunction, even after valve replacement [40]. This systolic dysfunction may also explain the significant late postoperative increase in the left atrium diameter in participants who undergo DSRS. In contrast, EGDS participants present with normal systolic function and do not have a significant increase in the left atrial diameter.

Hepatopulmonary syndrome (HPS) is characterized by a defect in the arterial oxygenation that is induced by pulmonary vascular dilatation brought about by liver disease [41]. In our study, IPV was detected in 9 participants (60%) after EGDS and in 9 participants (69%) after DSR (p > 0.05). This prevalence of IPV was greater than previously reported in patients with portal hypertension from hepatic cirrhosis (between 9% and 47%) [42,43,44,45]. This fact can partially be explained by the use of different diagnostic methods [46,47,48,49]. Some authors have used lung scintigraphy with labeled albumin, whose particle diameter is greater than 20 mm, while others have adopted the contrast-enhanced echocardiography with agitated saline solution [42,44,45,50,51] or indocyanine green, which favors the formation of bubbles larger than 90 mm, a diameter that is larger than normal lung microcirculation (8–15 mm) [43].

Some authors have adopted different diagnostic criteria for IPV that are based on the appearance of bubbles in the left atrium between the third and sixth [42,43,45,46] or fourth and seventh cardiac cycles [50,51]. In addition, most studies have only performed contrast-enhanced echocardiography in patients with hypoxemia or enlargement of the arterial-alveolar oxygen gradient, which certainly reduces the prevalence of IPV. We performed a contrast-enhanced echocardiography in all participants to determine the prevalence and effects of surgical procedure on IPV.

In fact, some authors [52,53,54] have identified the appearance of IPV during physical exertion in healthy people and have observed complete disappearance afterward. Therefore, in addition to vasoactive mediators [55,56,57,58,59] that are traditionally involved in IPV in liver cirrhosis, hyperdynamic circulation may be responsible for recruiting natural shunts located in the apical regions of the lungs and pleural surface [53,54] or directly causing vasodilatation in the pulmonary microvasculature [60,61]. However, the prevalence of IPV, independent of the surgical procedure used to treat portal hypertension, suggests that other factors, such as vasoactive mediators or collateral circulation, contribute to long-term changes in the pulmonary vascular tone.

It is also interesting to note the low incidence of HPS (one participant) detected in our study, especially when compared to previous data in portal hypertension from other etiologies [43,44,45,47,49,50,51,62]. The degree of intrapulmonary vasodilatation determines increased pulmonary blood flow, leading to an imbalance between the perfusion and oxygen diffusion, abnormal D (A—a) O_2_ gradient and the appearance of hypoxemia [43,63,64]. We hypothesize that patients with schistosomiasis have less marked vasodilatation than observed in cirrhosis from differences in the concentrations of vasoactive mediators, such as endothelin and tumor necrosis factor. Indeed, a recent study [64] identified lower serum levels of endothelin in patients with schistosomiasis than those observed in patients with cirrhosis [65,66].

Therefore, we report, for the first time, a prevalence of IPV in hepatosplenic mansonic schistosomiasis.

## Conclusions

The increase in the cardiac output and stroke volume associated with the increased volume and diameter of the left ventricular cavity, in the absence of valvular or left ventricular hypertrophy, demonstrated left ventricular dilation with preserved EF from an increase in the venous return in DSRS participants. EGDS participants present with long-term normal cardiac output and normal left ventricular function. Contrast-enhanced echocardiography revealed that the prevalence of IPV was greater than previously described in patients with PH from hepatic cirrhosis.

## Supporting Information

S1 Table Laboratory data of the participants with hepatosplenic schistosomiasis mansoni after surgical treatment for portal hypertension by distal splenorenal shunt (DSRS) and esophagogastric devascularization with splenectomy (EGDS). The results are expressed as the mean ± SD. ALT, alanine aminotransferase; AST, aspartate aminotransferase; GGT, gamma glutamyl transpeptidase; ALP, alkaline phosphatase; BUN, blood urea nitrogen; Cr, creatinine; TP, total serum protein; ALB, albumin; PT, Prothrombin time; PTT, Partial thromboplastin time; TB, Total bilirubin; IB, indirect bilirubin; Hb, Hemoglobin; Ht, hematocrit; WBC, white blood cells; PLT, platelets; *p < 0.01.
(DOC)

S2 Table Transthoracic echocardiography results in participants with mansonic schistosomiasis before (Preop) and after surgical treatment for portal hypertension by distal splenorenal shunt (DSRS) and esophagogastric devascularization with splenectomy (EGDS). The results are express as the mean ± SD. Preop, patients with portal hypertension due to hepatosplenic mansonic schistosomiasis before surgical treatment of portal hypertension; DSRS, Distal Splenorenal Shunt; EGDS, Esophagogastric Devascularization with Splenectomy; LA, left atrium; LVEDD, left ventricular end-diastolic diameter; LVEDV, left ventricular end-diastolic volume; LVESD, left ventricular end-systolic diameter; LVESV, left ventricular end-systolic volume; SF, shortening fraction; EF, ejection fraction; Se, septum wall thickness; PW, posterior wall thickness; *p < 0.0001; **p < 0.001; *** p < 0.05 between the DSRS and Control groups.
(DOC)

S3 Table Late postoperative hemodynamic parameters in participants with portal hypertension due to hepatosplenic mansonic schistosomiasis. The results are express as the mean ± SD. DSRS, distal splenorenal shunt; EGDS, esophagogastric devascularization and splenectomy; HR, Heart rate; MABP, mean arterial blood pressure; CO, cardiac output; SV, systolic volume; *p < 0.01 between DSRS and Control.
(DOC)
