# Supplementary material for: Left Ventricular Dilation and Pulmonary Vasodilatation after Surgical Shunt for Treatment of Pre-Sinusoidal Portal Hypertension
Source: PLoS One. 2016 Apr 27;11(4):e0154011. doi: 10.1371/journal.pone.0154011 (PMC4847763; doi:10.1371/journal.pone.0154011)
Supplement: S1 Table — The results are expressed as the mean ± SD. ALT, alanine aminotransferase; AST, aspartate aminotransferase; GGT, gamma glutamyl transpeptidase; ALP, alkaline phosphatase; BUN, blood urea nitrogen; Cr, creatinine; TP, total serum protein; ALB, albumin; PT, Prothrombin time; PTT, Partial thromboplastin time; TB, Total bilirubin; IB, indirect bilirubin; Hb, Hemoglobin; Ht, hematocrit; WBC, white blood cells; PLT, platelets; *p < 0.01. (DOC) [file pone.0154011.s001.doc]

**S1 Table. Laboratory data of the participants with hepatosplenic schistosomiasis mansoni after surgical treatment for portal hypertension by distal splenorenal shunt (DSRS) and esophagogastric devascularization with splenectomy (EGDS).**

The results are expressed as the mean ± SD.

ALT, alanine aminotransferase; AST, aspartate aminotransferase; GGT, gamma glutamyl transpeptidase; ALP, alkaline phosphatase; BUN, blood urea nitrogen; Cr, creatinine; TP, total serum protein; ALB, albumin; PT, Prothrombin time; PTT, Partial thromboplastin time; TB, Total bilirubin; IB, indirect bilirubin; Hb, Hemoglobin; Ht, hematocrit; WBC, white blood cells; PLT, platelets; *p < 0.01.
