# Supplementary material for: Left Ventricular Dilation and Pulmonary Vasodilatation after Surgical Shunt for Treatment of Pre-Sinusoidal Portal Hypertension
Source: PLoS One. 2016 Apr 27;11(4):e0154011. doi: 10.1371/journal.pone.0154011 (PMC4847763; doi:10.1371/journal.pone.0154011)
Supplement: S2 Table — The results are express as the mean ± SD. Preop, patients with portal hypertension due to hepatosplenic mansonic schistosomiasis before surgical treatment of portal hypertension; DSRS, Distal Splenorenal Shunt; EGDS, Esophagogastric Devascularization with Splenectomy; LA, left atrium; LVEDD, left ventricular end-diastolic diameter; LVEDV, left ventricular end-diastolic volume; LVESD, left ventricular end-systolic diameter; LVESV, left ventricular end-systolic volume; SF, shortening fraction; EF, ejection fraction; Se, septum wall thickness; PW, posterior wall thickness; *p < 0.0001; **p < 0.001; *** p < 0.05 between the DSRS and Control groups. (DOC) [file pone.0154011.s002.doc]

**S2 Table. Transthoracic echocardiography results in participants with mansonic schistosomiasis before (Preop) and after surgical treatment for portal hypertension by distal splenorenal shunt (DSRS) and esophagogastric devascularization with splenectomy (EGDS).**

The results are express as the mean ± SD.

Preop, patients with portal hypertension due to hepatosplenic mansonic schistosomiasis before surgical treatment of portal hypertension; DSRS, Distal Splenorenal Shunt; EGDS, Esophagogastric Devascularization with Splenectomy; LA, left atrium; LVEDD, left ventricular end-diastolic diameter; LVEDV, left ventricular end-diastolic volume; LVESD, left ventricular end-systolic diameter; LVESV, left ventricular end-systolic volume; SF, shortening fraction; EF, ejection fraction; Se, septum wall thickness; PW, posterior wall thickness; *p < 0.0001; **p < 0.001; *** p < 0.05 between the DSRS and Control groups.
