# Supplementary material for: Left Ventricular Dilation and Pulmonary Vasodilatation after Surgical Shunt for Treatment of Pre-Sinusoidal Portal Hypertension
Source: PLoS One. 2016 Apr 27;11(4):e0154011. doi: 10.1371/journal.pone.0154011 (PMC4847763; doi:10.1371/journal.pone.0154011)
Supplement: S3 Table — The results are express as the mean ± SD. DSRS, distal splenorenal shunt; EGDS, esophagogastric devascularization and splenectomy; HR, Heart rate; MABP, mean arterial blood pressure; CO, cardiac output; SV, systolic volume; *p < 0.01 between DSRS and Control. (DOC) [file pone.0154011.s003.doc]

**S3 Table. Late postoperative hemodynamic parameters in participants with portal hypertension due to hepatosplenic mansonic schistosomiasis.**

The results are express as the mean ± SD.

DSRS, distal splenorenal shunt; EGDS, esophagogastric devascularization and splenectomy; HR, Heart rate; MABP, mean arterial blood pressure; CO, cardiac output; SV, systolic volume; *p < 0.01 between DSRS and Control.
